# Observations on the Causes and Treatment of Ulcerous Diseases of the Leg

**Published:** 1835-04-01

**Authors:** 


*Observations on the Causes and Treatment of Ulcerous Diseases of the Leg.* By J. C. SPENDER, M.R.C.S.—London, 1835. 8vo. pp. 210.

WHEN an author prefaces his work by a statement that he has seen a thousand cases of the disease on which he writes, he must expect that such an announcement will produce a twofold effect upon his readers : for while, on the one hand, it commands an attentive examination of his opinions, it must, on the other, raise their expectations as to the interest and importance of the discoveries that have resulted from such extended experience. The offspring of such favourable opportunities of study on the present occasion is a method of treatment which must be considered rather as a modification of an old, than as any new line of practice. The author is sanguine as to its success, but we regret to say that he has not thought it worth while to narrate a single case in which it was employed. We do not mean in any way to distrust his assertions that his plan has been eminently serviceable in his own hands; but surgery is so essentially dependent upon facts, that we cannot conceive how any surgeon, who possesses cases to quote, can write a book without inserting at least a few to support his statements, and induce other practitioners to adopt his recommendations. One great distinction between the works of the long-established practitioner, who writes to instruct his fellows, and those of the unfledged youth, who writes for notoriety, is, that the one appeals constantly to facts which have occurred under his own observation, while the other relies on authority as the foundation of his reasonings. It would be absurd, as well as unjust, to enrol Mr. Spender in the latter class; for the accuracy of many of his distinctions, as well as the ease of his style, show a long familiarity with the subject. He is unquestionably a member of the more enviable class: we allow his claim to the estate, but are angry with him for refusing to exhibit his title-deeds. We do not know whether his discoveries were sufficiently numerous to make it necessary to embody them in an octavo volume; but, as authors and readers have long had different interests in the size of a work, we shall adopt the side of the latter, and, by confining our examination to such novelties as it contains, we shall at once do justice to our author's merits, and at the same time save our readers the trouble of perusing a quantity of matter with which they must be already acquainted.

The work is divided into four chapters: the first being devoted to the causes and classification of ulcers of the leg; the second, to the general principles of treatment; the third, to the application of these principles; and the fourth, to the question of the safety of healing ulcers. We shall make a few observations upon the first three chapters; but the question in the last has been so long settled, that we were not aware, till Mr. Spender's work came under our notice, that there was any one who would think it worth while at the present day to waste words upon the subject.

Our author commences by attempting to show that the dependence of the lower limbs, and their distance from the centre of the circulation, are not the common causes of ulceration of the legs. His argument, which is rather long, is founded upon the fact that everybody's legs are similarly situated: why then, he asks, does not everybody suffer? This would scarcely require an answer, if it were not that we are rather fond of proving that our forefathers were not absolutely fools; a doctrine which, it seems, every modern writer is anxious to establish. No one has, as far as we know, ever asserted that these circumstances were more than predisposing causes; and the experience of every modern surgeon will bear us out in the assertion that wounds more readily heal, *cæteris paribus*, in proportion to their proximity to the heart. But our author himself, in a subsequent part of the work, is willing to attribute to their position the frequency of varicose veins in the lower extremities, which varicose veins give rise to the majority of ulcers. This only places the dependence of the limbs one step further back in the scale of causes, so that the difference between his opinions and those of other surgeons is scarcely worthy of mention, much less of prolix argument.

The next point on which our author insists is, that varicose veins are not the accompaniments only, but the veritable causes, of the varicose ulcer; and he enters the arena against Mr. Benjamin Bell and Sir Everard Home, who do not, in his opinion, attach sufficient importance to their agency. But our author should remember that these are not the latest writers upon the subject, and that since their day our knowledge of the absorbing power of the veins has been much increased by the experiments of Magendie and others: nay more, there are surgeons, of high reputation too, who attribute the process of ulceration to the undue increase of this absorbent power.

The next point of importance touched upon by the author is the occurrence of irritable ulcers from varicose veins; and, as he entertains a rather novel opinion upon the subject, we shall quote his own words.

"Now it is not in the callous or indolent ulcer only that the varicose state of the veins prevails and pre-exists; it is very commonly met with in the irritable sore, and it is not easy to assign a reason why it should not here act a part equally important. If it produce any effect in the former case, it is not likely that it is harmless or inefficient in the latter. The mere circumstance of its forming at one time an ulcer of an indolent kind, is no reason why it should not form, at another time, an ulcer of an irritable kind; because we constantly see the same disturbing cause giving rise to different pathological effects, according to some modifying power in the constitution, or in the seat of the disease. Nor is it difficult, I think, to assign the reasons which appear to alter the operation of the one common cause, and which account for its giving rise to dissimilar effects. I have generally found that the varicose affection exists, more or less, in all kinds of very severe and refractory sores of the leg; and some of the worst states of this diseased condition of the veins I have ever observed were in conjunction with irritable ulcers. The circumstances which appear to determine whether this common condition of the veins shall exhibit itself in the production of an indolent or of an irritable kind of ulcer, are partly to be attributed to the constitution, and partly to local causes. The irritable ulcer is most frequently formed in persons of health and strength; whilst the indolent ulcer is most commonly produced in individuals advanced in life, or suffering from a weakened state of the system. Thus, men experience the irritable description of sore more frequently than women, and young persons more commonly than old. Then, with regard to the local modifying circumstances, I have almost always found that the indolent ulcer is situated more deeply than the irritable one; the former appearing to attack both the integuments and the less sensible cellular textures underneath, whilst the latter is chiefly confined to the vascular and excitable tunics of the skin itself. That this is the true explanation of the difference in the two instances, I am convinced, not only from having almost constantly observed a very bad ulcer of the leg, of either kind, to be preceded by a varicose condition of the veins, but still more decisively from having occasionally found, in the same individual, the same cause giving rise sometimes to one effect and sometimes to the other, according as the deeper or more superficial parts of the leg were affected. One of the most indolent and one of the severest irritable ulcers I have ever seen, were both situated on the same leg; the former placed more deeply in the substance of the limb, and the latter more superficially on its surface. Nor was this to be accounted for by supposing that the indolent ulcer was lower down the leg than the other, and thus farther from the centre of the circulation; for it happened that this ulcer was high up in the posterior part of the calf, whilst the irritable sore occupied the dorsum of the foot and front of the shin." (P. 18.)

This is a point on which Mr. Spender would have done well to have favoured us with cases. At present, the single case to which he alludes admits of a different explanation. Ulcers upon the instep are almost invariably irritable, even when not dependent upon varicose veins; and this evidently depends upon their situation, viz. upon the front of a joint which is constantly in action. We are therefore not prepared to accede to our author's hypothesis, that the irritability of ulcers depends upon their depth; though we quite agree with him in believing that irritable ulcers may be consequent on varicose veins. We are rather inclined to attribute this irritability, when it occurs, either to some peculiarity in the situation, as in the instance above mentioned, or more commonly to the state of the constitution. Every one must have seen indolent sores take on an irritative action, during some temporary disorder of the constitution, which has subsided when that disorder was removed. Of the relative number of irritable and indolent sores, our author has furnished us with the following table.

" Out of a hundred cases I have found

| | | | | |
|---|---|---|---|---|
| 79 Varicose, consisting of | 41 simple, | 27 very irritable, | 11 very indolent. | |
| 21 Non-varicose — | 15 ditto, | 4 ditto | 2 ditto. | |
| Of the whole number, | 68 females, | 32 males. | | |
| Of the 79 Varicose, | 59 ditto, | 20 ditto. | | |
| Of the 21 Non-varicose, | 9 ditto, | 12 ditto." | | (P. 29.) |

From the frequency of the varicose ulcer on which our author insists (and we think justly,) more than is generally done in books, he would derive his classification of ulcers. The following passage contains the method which he would wish to adopt.

" All ulcerous diseases of the leg appear to admit of a very easy and natural arrangement into general and local; and accordingly this has been adopted by almost every writer. The first, or constitutional class, is subdivided in conformity to the nature of the diseases from which the sores arise—as venereal, scrofulous, and the like. The subdivisions of the second or local class, instead of being derived, as is usually the case, from the mere external aspect of the ulcers, should, I think, be taken from the more important and permanent distinction arising from the presence or absence of the varicose state. All sores of this class may therefore be arranged under two orders, determinable by the condition of the veins,—first, varicose; second, non-varicose. Under each of these orders may be retained the species or distinctions arising from the superficial appearance and character of the ulcer, according to the threefold degree of action exhibited—irritable, simple, indolent. Thus we should have varicose irritable, varicose simple, and varicose indolent; and non-varicose, irritable, non-varicose simple, and non-varicose indolent. The two general classes may be further considered as separate or combined, according as the specific sore takes place on a varicose or non-varicose limb; giving, for instance, venereal varicose, venereal non-varicose, each possessing its threefold degree of activity; and so of the rest. The object of such a classification is to make the presence or absence of the varicose affection one of the leading foundations on which the whole is built, as being the element of the greatest practical importance. When I have observed this simple distinction in practice, I have in general found that it is comparatively of little consequence whether the superficial aspect of the ulcer be what is termed irritable or indolent, exuberant or callous. An attention to the varicose condition of the veins, when present, has, with nearly equal facility, removed each variety of ulcer, by the use of the same form of application hereafter described; whilst, on the contrary, the same application to the same kind of ulcer, so far as its mere external appearance is concerned, varies in its effects, just in proportion as it depends on the varicose state, unless great attention be directed to this constituent of the disease. Nothing can be clearer, therefore, that this last characteristic furnishes a much surer ground for a practical arrangement of ulcers than the former; or rather, that the former affords none at all.

"I believe all the ordinary arrangements of ulcers of the leg, taken from the mere superficial appearance and external character of the sores, are essentially defective. The inquiry should not be so much what is the outward aspect of the sores, as what is the inward state of the veins and other structures of the limb; as this last is chiefly to determine the plan and practice of treatment. I have commonly found that, if two ulcers of dissimilar external appearance result from this cause, nearly the same method of treatment will remove them; whilst, for the cure of two ulcers of the same superficial character, if the one arise from this cause, and the other not, a difference of management will often be required." (P. 52.)

We perfectly coincide in the propriety of our author's classification: it is evidently the result of experience, and leads to sound practical indications of treatment. We are, however, forced to dissent from his opinions with regard to the value of the appearances of ulcers in guiding our treatment. Every practitioner may not understand what these appearances indicate; but such as possess that knowledge must be too well aware of its utility, to surrender it at the command of any writer, however experienced.

The general principles of treatment recommended by our author are, 1st, in cases where the system is adequate to the removal of the disease, to imitate, as far as possible, the natural process; 2d, in other instances, we must endeavour to place the system in a position which will enable it to accomplish the cure; or, in other words, we must endeavour to produce a healthy action. For the former purpose, Mr. Spender recommends the formation of a scab over the surface of the ulcer, by means of an ointment, into the composition of which a large proportion of chalk enters. As this is the principal practical point of any novelty in the work, we shall quote the passage containing it.

"When the cure is left to nature, it seems to be only a superficial ulcer, which will readily scab; for, if it be deep, the quantity of matter is too great to be speedily evaporated into a crust. Now we can, in these cases, greatly assist the natural process by the addition of some harmless substance, which will thicken the discharge, and thus produce an incrustation; whilst, even in ulcers situated more on the surface, and which would sooner spontaneously scab, the same application will hasten the formation of the covering required.

"Of all the kinds of outward application which I have tried, an ointment containing a very large quantity of prepared chalk forms the best artificial crust. The earthy matter must be in a much greater proportion than enters into any ointment in the Pharmacopeia, consisting of about three pounds of chalk to two pounds of lard. Even four pounds of chalk will be readily taken up by two pounds of lard; and, if about three ounces of olive-oil be added, the ointment will not be too stiff, but will easily admit of being spread on the linen. The best method of preparing this application is not by rubbing the chalk down with the lard, but, having previously reduced the chalk to a very fine powder, heat the lard to a tolerable temperature, and, whilst it continues hot, gradually add the levigated chalk in the same vessel in which the lard was warmed. By this means it forms more of a solution than a mere addition, and the two ingredients thus become more intimately blended together. This should be stirred until it is nearly cold, and then placed by for use. I have found that preparing the ointment in this manner is preferable to the more usual mode of making such substances, by simple admixture or trituration, as it produces a mass more homogeneous, containing less of the earthy particles in a distinct or separate form." (P. 64.)

From the use of this ointment the author proposes four advantages.

"1. It is extremely mild and harmless in its nature, so that it very rarely produces pain. Out of several hundreds of cases in which I have employed it, I have scarcely known more than two or three in which it did not give great and immediate ease; and in these exceptions there were other circumstances existing to account for the fact. I have frequently heard patients exclaim how comfortable their legs have felt as soon as any irritating application has been thrown aside, and the chalk-ointment applied to the sore. The mildness of this application is probably owing to the large quantity of the alkali, which is ready to unite with the acid generated by the animal matter, on the presence of which rancidity appears to depend; and thus the lard is prevented from running into this state. It is well known that most of the common ointments soon become rancid by keeping, and that they are then very irritating to a sore. The chalk-ointment however never spoils, but retains its freshness for any length of time.

"2. As soon as the lard is melted by the heat of the part, and absorbed by the bandage, (and no more is employed than sufficient to hold the earthy matter in combination,) the chalk is disengaged, and a portion of it combines with the discharge. This fluid, in many ulcers, is extremely acrid; inflaming and excoriating the skin in the neighbourhood, and perhaps, on the principle of inoculation, perpetuating the disease. Some of the chalk unites with this secretion, and, greatly depriving it of its irritating properties, converts it into a comparatively harmless compound.

"3. Partly from this compound, and partly from other portions of the chalk itself, an incrustation is formed, first on the surrounding skin, then on the margins, and at last on the face of the ulcer. This crust is produced in a very gradual manner; and, when the discharge is great, sometimes none is deposited for the first two or three dressings. It is this slow method of its formation that constitutes its excellency, as in this respect it resembles the concretion of the natural scab. * * *

"4. From all these circumstances combined another great advantage is obtained. The dressings are not required to be removed so frequently, and the surface of the ulcer is consequently less exposed to disturbance. The mildness of the application, together with its neutralizing powers, lessens the pain; whilst its union with the discharge, or its more direct deposit, forms a harmless crust, to shield and protect the adjoining tender skin. The consequence is, that the dressings will remain on for a much longer period without producing uneasiness than when some other descriptions of ointment are employed." (P. 65.)

The formation of the scab is much more gradual, according to our author's experience, when the chalk is thus precipitated from an ointment, and its surface is more even and better modelled to that of the sore, than when the powder is sprinkled on the part.

In cases of blisters and burns, where the cutis vera is not destroyed, this latter plan has been long in use; but we doubt whether it may not give place with advantage to that proposed by our author.

There are four circumstances, however, which may prevent the cicatrization of the ulcer under the use of this ointment:

viz. 1st, varicose veins; 2d, adventitious deposits; 3d, the form of the ulcer; 4th, the existence of constitutional disturbance; and it is in these cases that the second general principle before mentioned must be brought into action. This is effected, in the first two instances, by the employment of compression.

The views which our author entertains of the method in which pressure is useful to a limb, are very sound. Mr. Baynton, whose name must be honourably mentioned whenever the treatment of ulcers is in question, did not sufficiently insist upon the value of the general compression to the subcutaneous structures, but appeared to attribute its principal efficacy to its approximating the edges of the ulcer. Mr. Spender, on the other hand, distrusts the benefit derivable from this source. For our own part, we hold a middle course between these two writers. We have frequently seen the application of a few short strips of plaster across the sore greatly expedite its cicatrization, though at the same time we placed our principal reliance on the general pressure of a bandage. The support of the venous circulation, and the stimulus to the absorbents to remove any adventitious deposit, are undoubtedly the most important advantages of compression, and our author has pointed out this fact with great distinctness at the conclusion of the chapter on the general principles of treatment.

The method in which these principles are to be applied forms, as we have said, the third chapter, which commences with a philippic against poultices, which our author would entirely banish from our store of remedies. His arguments, as might be expected, are far from strong, and we shall therefore reply to them no further than by assuring him that poultices are not only useful when the parts are sloughing, but when there is considerable inflammation, and but little discharge: where there is tension around the edges of the wound, attended by pain, there is no remedy that gives so much relief as this despised poultice. Where different stimulants have been applied for a long time, till the base of the sore has become hard, and its surface callous to their action, the use of a poultice for a few days will soften the parts, and restore their previous excitability.

It seems to us that our author is too sanguine in his temperament, for he is never convinced that a remedy is good but he thinks it necessary to denounce its opposite as bad. With much truth he asserts that ulcers will heal during the employment of exercise, provided that a tight bandage be worn round the limb; but this is not sufficient,—he must prove that rest is prejudicial. From long experience in the treatment of old ulcers of the leg, we have come to this conclusion, that they will heal more expeditiously where the horizontal position is preserved, than where the patient is allowed to take exercise; but that in the former case they are more liable to give way than in the latter.

We cannot pass over page 100 without censure. A note is attached to it, which looks like a close imitation of the paragraphs that have for several years sullied some periodical medical publications. We can assure Mr. Spender, that rank in a liberal profession is not to be obtained by the depreciation of others, nor by encouraging pupils to criticise and arrogate superiority to their teachers. Should his work reach a second edition, we trust that this note will be expunged. Here is the offending spot.

"I trust it will not be imputed to any unworthy motive, if I quote, in confirmation of this statement, an extract from a letter, written by an observant young man, who has lately left me to attend the hospitals in London: 'I took the opportunity of being present the other day, when ——, one of the surgeons, was prescribing for the out-patients, and, as is always the case, there happened to be plenty of ulcerated legs. Among the rest, a poor woman unrolled a large poultice from a varicose ulcer, which, by the by, I would have cured in a month. ——'s remarks were much as follow: "Gentlemen, this ulcer is plainly produced from a varicose state of the veins: now, nothing will do in these tedious cases but rest; rest, gentlemen, is the grand desideratum." The woman's reply was, that she could not possibly afford to lie in bed, as she had a family to support. Then —— told her, "Your leg will never get well, but you shall try some fresh application." He accordingly prescribed the black wash, which he appears to make use of in all those cases which are not poulticed. I am afraid ——'s treatment is too general.'"

We now come to our author's plan of dressing the sore, which we shall allow him to describe in his own words.

"The first thing is, to cover the surface and sides of the sore for some distance beyond its edges with the chalk-ointment, spread about the thickness of a wafer on thin linen. I think the linen is preferable to lint, as it seems to allow the disengagement of the chalk more readily. No compresses of any sort are placed on this, as I am convinced the frequent use of paddings of linen, calico, and the like, placed between the dressing and the roller, are prejudicial, by unnecessarily loading and heating the part, and by confining, or preventing the escape of the matter." (P. 114.)

Here follow some very minute directions for the application of a bandage. The author is in the habit of applying his bandages very tight. The best rule we believe to be, the tighter the better, provided that they do not produce pain. But to proceed with his directions for dressing the sore.

"The length of time which elapses before the bandage and dressings are removed and reapplied, must, of course, be regulated by the circumstances of the case. When the ulcer is very extensive, and the discharge proportionally great, it will be necessary to dress the leg, perhaps, every day, at the commencement of the treatment." (P. 134.)

"The object is, to delay the removal as long as possible, without, however, carrying this forbearance too far; and if, instead of measuring the time by the mere number of hours or days, we take the surer criterion of the presence or absence of uneasiness, we shall be in no danger, on the one hand, of acting too soon, or, on the other, of waiting too long.

"It is true that sometimes the quantity of discharge is so considerable, and becomes so offensive, that common cleanliness demands the frequent renewal of the dressings. In such instances it will be commonly found that some uneasiness also is felt by the patient; but, even if this should not be experienced, the laudable wish, on his part, of being kept as free as it may be possible from filth and stench, must be obeyed, and the dressings may then be removed, without waiting for the presence of any other inconvenience." (P. 136.)

"The bandage being undone, the thin linen dressing is raised very gently, in order to allow as much of the chalk-ointment to be left behind as may be attached to the surrounding parts and surface of the sore. We shall frequently find, even after the first dressing, that a thin film is formed about the margin, especially at the upper side, however large the ulcer may be; and, if it be a small one, in addition to the deposit on the edges, some of the incrustation may probably be observed on the face of the sore. This must not by any means be taken off; it is treason to touch the chalky scab." (P. 137.)

"At each succeeding dressing, we commonly shall be able to observe a gradual increase of the chalky incrustation, first on the sides of the ulcer, then on its margin, and by degrees over its surface. Before, therefore, it covers the sore, it is placed all over the bordering integuments, which is a circumstance of very great importance. Every one must have seen that sometimes the surrounding skin becomes excoriated, and even ulcerated, by the discharge; and this, as has been already alluded to, is one cause which renders the frequent removal of the dressings necessary. As soon, however, as the chalky crust is formed, it effectually shields the delicate skin from the presence and irritation of the matter, the fluid passing down over the incrustation without touching the neighbouring integuments. The skin is thus protected from the exciting properties of the purulent or other fluid, and being likewise shut in from any external influence, becomes firm, substantial, and sound. There is no danger, as I have already stated, that the chalky crust will form over the ulcerated part, and become a barrier to the escape of the fluid. A layer of powdered chalk in substance, or a thick watery solution of it, will indeed produce an impervious coating, and confine the discharge; but the slow and gradual manner in which the artificial scab is deposited by the use of the ointment, renders this evil unlikely. So long as the discharge continues considerable, it is enough to wash off the thin film left by the chalk-ointment, and to prevent its attachment to the face of the sore; and it is only as the discharge diminishes that the crust is completed." (P. 139.)

"When the ulcerated surface is gradually covered by the chalky crust, and its whole extent is included, it should not be disturbed until a sufficient time has been allowed for the entire part to heal underneath. I have been struck with surprise to see how rapidly this is effected, when thus shut in and protected, as Nature herself would do it; but still an idle curiosity to see how things are going on below should never be indulged. As soon as the ulcer is completely healed, the artificial scab becomes hardened, from the want of the moisture from the discharge; it then cracks, and, separating into pieces, comes off by degrees itself." (P. 142.)

Such are the details of the plan proposed by the author. It certainly appears feasible; but we have never tried it, and the book contains no cases of its success. It is but just to say, that Mr. Spender has entered so minutely into the subject, that we have no doubt of his having employed the method extensively. We can only say, that we intend to give it a trial.

The remainder of the chapter is devoted to observations on the modifications of this plan, required in certain states of the ulcer: they are, however, little else than the ordinary rules for the dressing of irritable and unhealthy ulcers, which it would be a work of supererogation to present to our readers. But the following observations are so good, and, as far as we know, not generally known, that they deserve to be an exception to our rule.

"Whilst, however, the general indication for the employment of compression is as discernible in the majority of irritable ulcers as in the other varieties, there are occasionally existing circumstances which interfere with the full application of the principle to practice. Sometimes the ulcerated part is so tender, and excruciatingly painful when touched, that it will scarcely bear anything on its surface; and in such instances it would be cruelty to apply a tight bandage over it. In these cases, before they are made better, and able to submit to compression, very great benefit may be derived from using the roller in the following manner: Let the bandage be applied as tightly as usual, from the root of the toes up to the lower margin of the sore; and, when it has arrived at this point, it can be secured, and placed on more slackly around the remaining part of the limb, so as not to give pain by its local pressure on the ulcer. The support which is given to the vessels below greatly improves the character of the sore: it soon becomes altered in appearance, and, what is of great importance, considerable relief from severe suffering is commonly felt. By this means, though prevented from obtaining the full advantage of the bandage at once, from the sensitive nature of the ulcer, much of its benefit is nevertheless gained; and the diseased part is soon converted into a state which will bear the more complete use of the remedy. At other times the irritable sore, though not so intensely painful, may be situated on a part of the leg which makes it incapable of bearing the bandage to its proper degree of tightness. One very common place which such ulcers occupy is the front of the leg, running up the shin; and such parts will not endure to be pressed by the bandage on the hard tibia immediately underneath. We must then put the roller on the foot and around the ankle, according to its usual degree of force, until we approach the seat of the evil; and, having arrived there, we must allow the bandage to describe the other parts of its course without being so tight. When, therefore, either from the severe pain, or from the local position of the irritable ulcer, the patient cannot bear the full pressure at once, we must be satisfied to compromise matters a little, and proceed more slowly in our efforts. This, however, is very different from abandoning the principle, losing sight of the origin of the disease, and, forgetting the indication thus afforded, using fomentations and poultices. Strictly speaking, indeed, such modified use of the bandage, gradually increased up to its proper extent and power, can hardly be called an exception to its use: it is only a temporary limitation of its application, resulting from circumstances associated with the disease." (P. 166.)

We have now given our opinion freely on the merits and demerits of the work. The latter are such as may be easily removed by the publication of a few cases, and the insertion of a few horizontal scratches in the manuscript: the former will remain evidences of Mr. Spender's industry in collecting facts, and talent in commenting upon them.

